# A pcu topology metal–organic framework, Ni(1,4-bib)(inca)_2_, that exhibits high CO_2_/N_2_ selectivity and low water vapour affinity†

**DOI:** 10.1039/d5ta01995h

**Published:** 2025-04-29

**Authors:** Samuel M. Shabangu, Alan C. Eaby, Sousa Javan Nikkhah, Lilia Croitor, Tao He, Andrey A. Bezrukov, Matthias Vandichel, Michael J. Zaworotko

**Affiliations:** a Department of Chemical Sciences, Bernal Institute, University of Limerick Limerick V94 T9PX Republic of Ireland xtal@ul.ie

## Abstract

Herein we report the synthesis of a new metal–organic framework, Ni(1,4-bib)(inca)_2_ or pcu-1-Ni, where 1,4-bib = 1,4-bis(imidazole-1-yl)benzene, inca = indazole-5-carboxylic acid, through the crystal engineering strategy of using an N-donor linker to pillar a square lattice, sql, topology net. pcu-1-Ni adopts pcu topology and features two types of hydrophobic pore, small pore A and large pore B. The biporous nature of pcu-1-Ni is reflected in its stepped CO_2_ and H_2_O adsorption isotherms, highlighting the influence of pore size and chemistry on gas and water vapour sorption properties. pcu-1-Ni exhibits the unusual combination of high CO_2_/N_2_ selectivity (IAST selectivity 100–250) and low water affinity at low RH (an S-shaped water vapour isotherm with an inflection point at 45–65% RH). Whereas pcu-1-Ni degrades upon repeated exposures to water vapour, its structure–property relationships can provide guidance for design of the next generation of CO_2_-selective sorbents. In this context, Canonical Monte Carlo simulations provide insight into the preferential adsorption of CO_2_ over N_2_ and H_2_O.

## Abstract 2

A porous coordination network with two types of ultramicropore offers a rare combination: strong CO_2_ binding and weak water affinity at <50% RH. Structural and modelling studies provide insight into the binding sites responsible for this behaviour.

## Graphical abstract

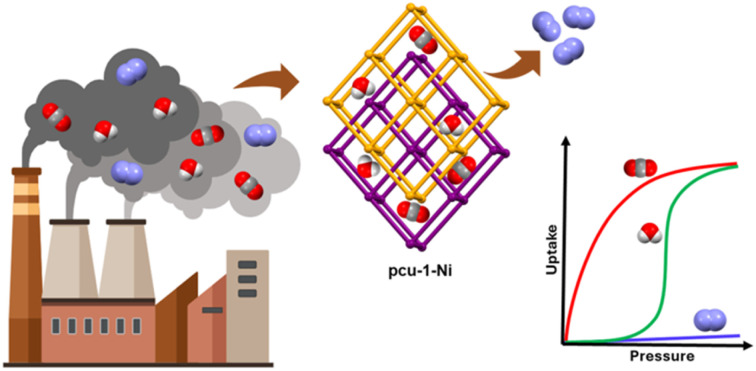

## Introduction

Metal–organic materials (MOMs),^1,2^ including metal–organic frameworks (MOFs)^3,4^ and porous coordination polymers (PCPs),^5,6^ are a class of porous materials that have attracted attention due to their amenability to design based on crystal engineering^7,8^ principles. This strategy has enabled the development of MOFs with potential utility for direct air capture of CO_2_,^9,10^ separation of CO_2_ from N_2_ (*e.g.* flue gas remediation),^11,12^ atmospheric water harvesting^13,14^ as well as indoor humidity control (IHC).^15,16^ Flue gas remediation is often mitigated by the presence of other components in gas feeds.^17^ For example, the presence of water vapour in downstream feedstocks poses a challenge because water adsorption can overwhelm CO_2_ affinity in MOFs,^18^ even in the most CO_2_ selective sorbents, such as hybrid ultramicroporous materials, HUMs.^19^ This is largely because most binding sites that are highly selective for CO_2_ over N_2_ also bind to H_2_O.^20^ Therefore, designing MOFs with high CO_2_ selectivity while controlling water adsorption remains a critical design challenge in crystal engineering.

The node-and-linker approach to design of coordination networks introduced by Hoskins and Robson^8^ provides an effective strategy to crystal engineering (design) of structural motifs that are suitable for the control and fine-tuning of properties. This approach offers several prominent structural motifs with square lattice (sql, 2D)^21^ and primitive cubic (pcu, 3D)^22^ topologies being amongst the most prevalent families of coordination networks.^23^ Many pcu networks are constructed from mixed-linker systems, where two (or more) linkers are integrated within a structure, thereby providing much more compositional diversity than is possible with a single linker.^24,25^ Commonly occurring networks are those comprised of sheets with composition ML_2_ (L = linker ligand) pillared by inorganic^26^ or ditopic N-donor linkers,^27^ L′, resulting in 3D networks of formula ML_2_L’. An archetypal example of the former is SIFSIX-1-Zn,^28^ a HUM comprised from sql layers pillared by hexafluorosilicate (SiF_6_^2−^, “SIFSIX”) dianionic linkers.^26,28,29^ By systematically varying the inorganic pillar, second generation materials with improved properties such as NbOFFIVE-1-Ni (ref. 30) and TIFSIX-3-Ni (ref. 31) were readily generated. Another commonly exploited type of pillar is represented by N-donor linker ligands such as pyrazine and dipyridyl-type linker ligands (*e.g.* 4,4′-bipyridine).^32,33^ The prototypical example of such “pillared-layered” MOFs is DMOF-1,^32^ in which the dicarboxylate linker, 1,4-benzenedicarboxylate, forms sql layers from paddlewheel dinuclear Zn_2_ units that are pillared by diazabicyclooctane to generate the resulting pcu topology MOF. By varying the organic N-donor pillar linkers, numerous variants of DMOF-1 have been developed such as MOF-508 (ref. 34) and X-pcu-*n*-Zn (ref. 33) (X = extended ligand or “X-ligand”), examples of which have demonstrated potential utility in hydrocarbon separation.^35^

The linker ligands used for pcu frameworks not only affect pore chemistry and sorption properties, they can also facilitate interpenetrated or non-interpenetrated structures.^36^ Interpenetration can be enabled by the use of longer linkers, and necessarily reduces porosity.^36,37^ Conversely, when one or both linkers are relatively short, this can reduce the level or even the existence of interpenetration.^36^ Nevertheless, reduced porosity does not necessarily equate to poor properties as ultramicroporous interpenetrated materials can offer enhanced gas separation properties that surpass the performance of non-interpenetrated analogues as seen for SIFSIX-2-Cu and SIFSIX-2-Cu-i (i = interpenetrated).^36,38^

N-donor linker ligands are not limited to those based upon pyridyl moieties. 1,4-Bis(imidazole-1-yl)benzene, 1,4-bib, is of interest to us and others as it can adopt both *cis* and *trans* configurations, which result in unusual network structures and enhanced sorption properties.^39–42^ 1,4-Bib can also afford 3D porous structures with dia (ref. 40 and 41) or pcu (ref. 42 and 43) topology, both being widely studied platforms for the evaluation of sorption properties.^44,45^ However, the use of 1,4-bib in the construction of 3D pcu MOFs remains underexplored. Indeed, our survey of the 3D MOF subset of the Cambridge Structural Database (CSD), focusing on 1,4-bib coordinated to transition metal ions, identified 238 3D network structures with pcu topology, among which only 34 exhibit potential porosity. Notably, the water vapour sorption properties of these MOFs have not been evaluated, including key performance parameters such as sorption kinetics and recyclability, representing a significant gap in the study of these pcu topology MOFs.

**Fig. 1:**
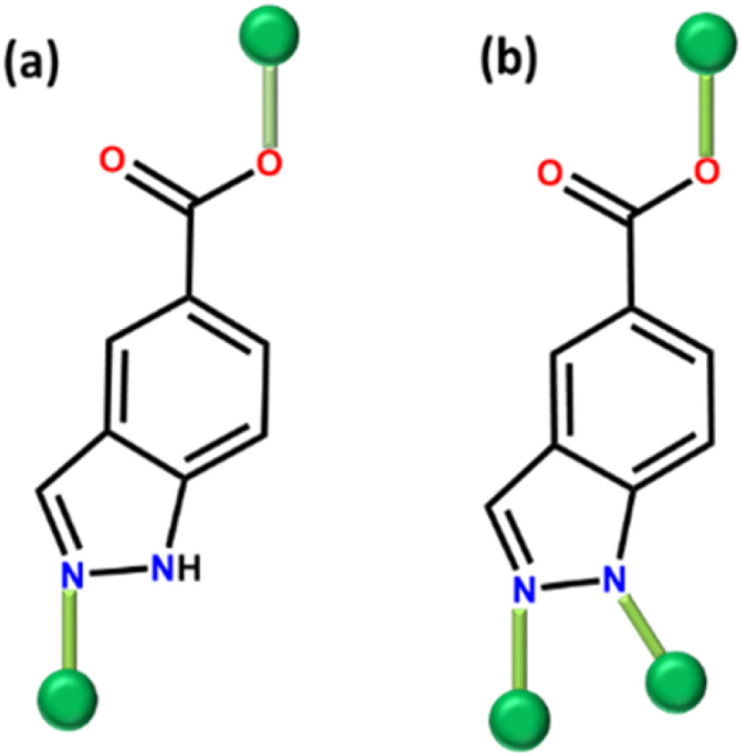
Schematic representation of coordination modes of (a) (inca)^−^, (b) (inca)^2−^.

Herein we report on the use of 1,4-bib with indazole-5-carboxylic acid (inca). The latter features both carboxyl and azole groups and has been previously used for the design of sql layers that can be pillared into pcu MOFs.^46^ H_2_inca can exist as either a monoanionic linker (Fig. 1a) or a dianionic linker (Fig. 1b), facilitating the construction of pcu architectures when paired with pillaring N-donor linkers as exemplified by {[Zn(inca)(pbptz) and Zn(inca)(4,4-bipy)], pbptz = 3,6-bis(4-pyridyl)-1,2,4,5-tetrazine, 4,4-bipy = 4,4-bipyridine}.^46^ Our survey of the MOF subset of the CSD revealed only 10 hits based on inca, 6 of which form sql networks when inca is a monoanionic linker (REFCODES: RUHKEN, RUHKEN01, ZIJHUZ, ZIJJEL, NUYNED and SEGBIR)(Table S1†). When present as a dianionic linker, inca can facilitate the creation of 3D porous structures as a single linker (REFCODE: SEGBOX and ELENOB) or sql layers pillared by N-donor linkers (REFCODES: BUTZUO and BUVBAY) (Table S1†). To date, previous reports based on this ligand have addressed gas sorption properties (Table S1†).^46–49^ Analysis of these studies revealed that only high pressure 273 K CO_2_ isotherms have been reported in BUTZUO and BUVBAY (Table S1†). To the best of our knowledge, no studies have reported combination of inca and 1,4-bib to construct 3D ‘pillared sheet’ platforms and evaluate their gas and water vapour sorption properties, the matter we address herein.

## Results and discussion

**Fig. 2:**
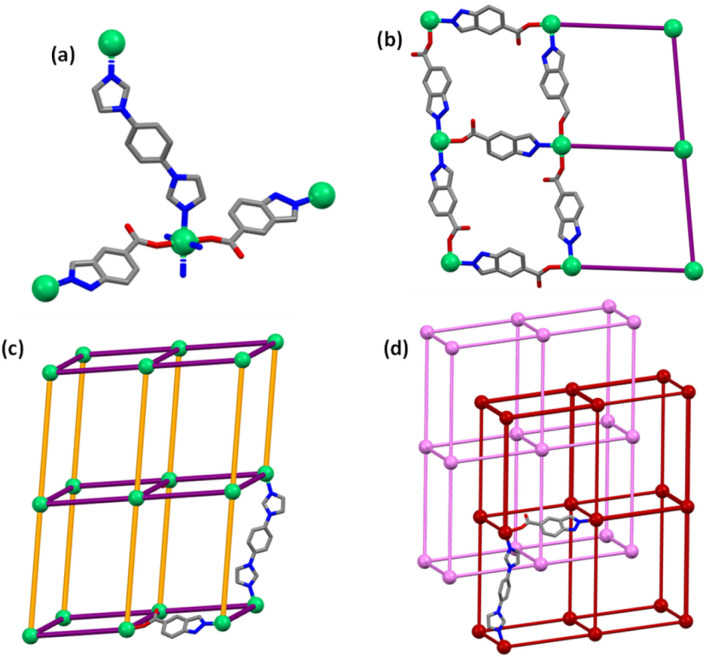
(a) asymmetric unit, (b) 2D square grid, (c) 3D porous structure and (d) 2-fold interpenetrated pcu-1-Ni. (Colours: orange = 1,4-bib, purple = inca, pink and dark red represent the two independent interpenetrated pcu structures). Some atoms have been omitted for clarity.

Solvothermal reaction of 1,4-bib, inca and Ni(NO_3_)_2_·6H_2_O afforded green block-shaped single crystals of the as-synthesised form, pcu-1-Ni (see ESI† for Experimental details). Single-crystal X-ray diffraction (SCXRD) experiments revealed that pcu-1-Ni had crystallised in the monoclinic space group *P*2_1_/*c* (Table S2†). The asymmetric unit is comprised of two (inca)^−^ ligands and one 1,4-bib ligand (Fig. 2a). The structure can be described as an sql network formed by Ni(ii) atoms linked by monoanionic inca ligands (Fig. 2b) that is pillared by 1,4-bib linker ligands to form a 3D pcu topology framework (Fig. 2c) with 2-fold interpenetration (Fig. 2d). Along the *c*-axis, there are two types of ultramicropore, A and B, with effective pore windows of 3.0 Å × 6.8 Å and 5.3 Å × 5.8 Å, respectively, and a calculated guest-accessible void volume of *ca*. 33.7% (Fig. S1†). SCXRD analysis revealed that pore B is occupied by *N*,*N*-dimethylformamide (DMF) and H_2_O. (Fig. S1†). Phase purity was supported by comparison of the calculated and experimental PXRD diffractograms (Fig. S2†). Thermogravimetric (TG) analysis (Fig. S3†) revealed that solvent loss occurred below 500 K. A methanol exchanged sample of pcu-1-Ni (3 × 72 h) revealed 11% weight loss at an onset temperature of 353 K. *In situ* variable temperature powder X-ray diffraction (VT-PXRD) measurements demonstrated that heating pcu-1-Ni under N_2_ flow did not induce a phase change, consistent with structural rigidity (Fig. S4†).

### Gas sorption and *in situ* PXRD

**Fig. 3:**
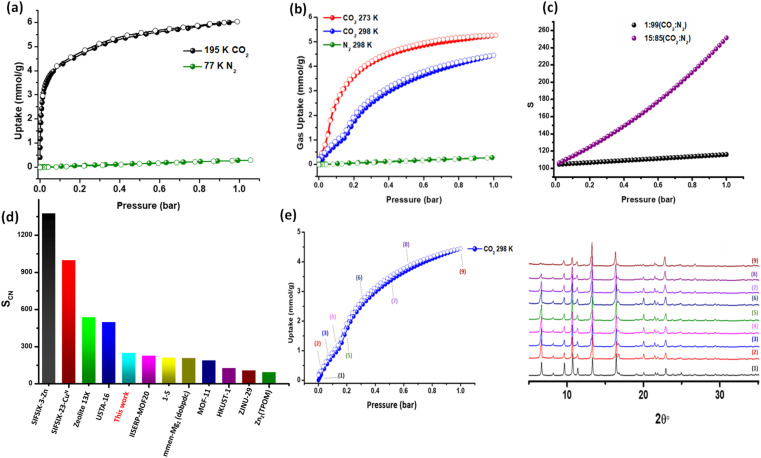
CO_2_ and N_2_ sorption isotherms at (a) 195 and 77 K (b) 273 and 298 K (c) IAST selectivities at 298 K for CO_2_/N_2_ (1 : 99 and 15 : 85) plotted as a function of pressure (d) comparison of CO_2_/N_2_ IAST selectivities with well-studied materials (15 : 85) and (e) *in situ* PXRD at variable CO_2_ pressure at 298 K.

To evaluate gas sorption properties, a methanol-exchanged sample of pcu-1-Ni was activated at 373 K under dynamic vacuum for 10 h. We conducted CO_2_ (195 K) and N_2_ (77 K) gas sorption measurements and pcu-1-Ni revealed CO_2_ uptake of 6.02 mmol g^−1^ at 1 bar and negligible uptake of N_2_, an indication that it could be a potential CO_2_/N_2_ selective sorbent (Fig. 3a). The CO_2_ sorption isotherms at 273 and 298 K revealed a step in the adsorption branch of the isotherm. Specifically, CO_2_ sorption at 273 K follows a Type-I^50^ isotherm profile until 0.05 bar, with CO_2_ uptake of 0.7 mmol g^−1^, followed by an inflection and subsequent sorption of 5.3 mmol g^−1^ at 1 bar (Fig. 3b). Similarly, at 298 K an initial Type-I isotherm profile is observed, with step onset at 0.18 bar and CO_2_ uptake of 1.1 mmol g^−1^, reaching a maximum uptake of 4.5 mmol g^−1^ at 1 bar (Fig. 3b). Negligible N_2_ uptake at 298 K was observed (Fig. 3b). A comparison with literature reported values revealed that the gravimetric uptake of pcu-1-Ni at 1 bar is among the highest in the context of CO_2_ sorbents (Table S3†). The selectivity of pcu-1-Ni for CO_2_ suggested to us the possibility of separating CO_2_ from N_2_. Whereas ideal absorbed solution theory (IAST)^51^ calculations based upon 298 K single-component adsorption isotherms can serve as an indicator of separation performance, care must be taken in the case of stepped isotherms. Nevertheless, IAST calculations afforded selectivities of 250 (15 : 85) and 118 (1 : 99) at 298 K and 1 bar for CO_2_/N_2_ (Fig. 3c and Table S4†), based on the initial slope of the 298 K isotherm. This selectivity is relatively high among CO_2_/N_2_ selective sorbents at 15 : 85 composition (Fig. 3d) but lower than benchmark sorbents. To better understand the CO_2_ isotherm profile, *in situ* variable pressure PXRD measurements were performed under CO_2_ at 298 K. The PXRD diffractograms remained unchanged upon exposure to CO_2_ up to 1 bar (Fig. 3e), indicating that no phase change had occurred. This suggests that the pcu-1-Ni retains its structure and does not undergo any significant structural rearrangement or swelling induced by CO_2_, indicating that there are multiple CO_2_ binding sites.

### Water vapour sorption

**Fig. 4:**
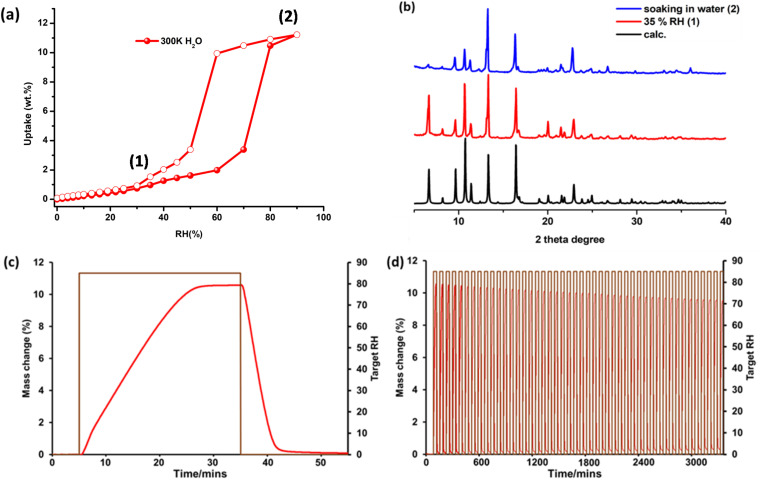
(a) Water vapour sorption isotherm recorded on pcu-1-Ni at 300 K; (b) calculated (calc.) and experimental PXRD diffractograms collected at various RH values; (c) water vapour sorption kinetics and (d) 50 humidity swing cycles (0–85% RH) on 9 mg of sample (300 K).

To evaluate water vapour sorption properties, dynamic vapour sorption (DVS) experiments were conducted at 300 K on a 9 mg sample of pcu-1-Ni and they revealed an S-shaped isotherm profile. The isotherm exhibits gradual uptake reaching 2 wt% at 55% RH with a step between 55 and 65% RH, resulting in uptake of 11 wt% at saturated humidity (Fig. 4a). Moderate hysteresis upon desorption was observed. The shape of the isotherm profile (*i.e.*, S-shaped at 45–65% RH range) is in accordance with the recommended working humidity range for IHC (45–65% RH) to ensure comfortable moisture levels in an indoor environment. This kind of S-shaped water vapour sorption isotherm, wherein the adsorption and desorption branches are centred between 45 and 65% RH, has only been reported in a few sorbents, namely rigid MOF-101,^52^Cr-soc-MOF-1,^53^UiO-67,^16^Y-shp-MOF-5 (ref. 15) and flexible Znbtca (ref. 54) and sql-(azpy)(pdia)-Ni.^55^ To gain a deeper understanding of the nature of the water vapour sorption, an attempt was made to perform H_2_O solvent exchange using as-synthesized crystals suitable for SCXRD analysis. Unfortunately, the crystals underwent a reduction in crystallinity after soaking for 3 days at 308 K, rendering them unsuitable for SCXRD analysis. Alternatively, analysis of PXRD patterns after exposure at various levels of RH was conducted. As revealed in Fig. 4b, incubation of an activated powder sample of pcu-1-Ni in a 35% (1) RH chamber for 48 h and soaking in H_2_O (3 × 2 days) (2) did not result in any changes in the PXRD pattern, further suggesting structural rigidity and some degree of hydrolytic stability. Overall, these data indicate that the S-shaped water vapour isotherm profile is consistent with a pore-filling^56^ (type V) mechanism that is typical of rigid water sorbents.^57^ The sorption kinetics of pcu-1-Ni were studied by subjecting a sample (9 mg) to humidity swing conditions at full loading and unloading (0 to 85%). As shown in Fig. 4c, pcu-1-Ni underwent 28 min of adsorption followed by 15 min of desorption. The slow adsorption rate for pcu-1-Ni is expected as kinetics of adsorption is often dependent on the position of the inflection point in the water vapour isotherm, as explained by our recently reported sorption kinetics isotherm determination (SKID) model.^58^ To evaluate recyclability, humidity swing conditions at 300 K (0–85% RH) were applied to a 9 mg sample of pcu-1-Ni for 50 cycles of 43 min each (28 min adsorption, 15 min desorption). Unfortunately, pcu-1-Ni exhibited a loss of working capacity during the test (11.3 wt% at cycle 1 *vs.* 8.6 wt% at cycle 50) (Fig. 4d).

### Computational studies

To better understand the gas and water vapour sorption behaviour in pcu-1-Ni, we employed Canonical Monte Carlo (CMC) simulations and Grand Canonical Monte Carlo (GCMC) simulations. The CMC experiments generated density maps that highlight the probable adsorption sites for CO_2_, N_2_, and H_2_O within the framework (Table S6 and Section S9†). These simulated probability density maps reveal sorbate–sorbent interactions between in both pores A and B (as represented in Fig. S6†). However, the larger pore B features the most plausible adsorption sites for all adsorbates, therefore, it is no surprise that CO_2_ and H_2_O exhibit stepped isotherms, suggesting both pore types are filled sequentially during sorption (see Movies S1–S3†).^59^ In contrast, in both pores, the distribution density of N_2_ is lower than CO_2_ at 1 bar and 298 K, demonstrating a weaker adsorption affinity for N_2_ (Tables S6 and S7†). This trend is also reflected in the experimental and GCMC simulated adsorption isotherms (Fig. 3b and S7†), further corroborating the experimentally observed selectivity. The high CO_2_ uptake can be attributed to the strong adsorption binding sites (see Table S7†). The higher CO_2_ uptake observed at 273 K compared to 298 K (see Fig. 3b and S7†) can be attributed to the exothermic nature of adsorption, where lower temperatures favour stronger interactions between CO_2_ molecules and the framework. The preferential adsorption of CO_2_ can be attributed to its higher quadrupole moment and polarizability, which facilitate stronger electrostatic and van der Waals interactions with the framework.

In the case of water, the hydrophobic nature of the framework results in minimal water adsorption at low RH, as the pore surface offers limited interaction with water molecules. However, as RH exceeds 55%, water molecules begin to form clusters *via* hydrogen bonding. Pore B, with its more accessible geometry, is filled first as water molecules preferentially occupy this pore. Once pore B is filled, water proceeds to occupy the more confined pore A. These clusters facilitate the adsorption of larger aggregates, enabling water to penetrate the confined pores (both A and B) of the framework. Despite the hydrophobic surface, water's small kinetic diameter (2.65 Å) allows it to access these pores, and the aggregation of water molecules is energetically favourable. This resulted in the steep uptake observed in the isotherm at >55% RH (Fig. 4a), illustrating that even in a moderately hydrophobic environment, water can exploit the framework's pore geometry.

**Fig. 5:**
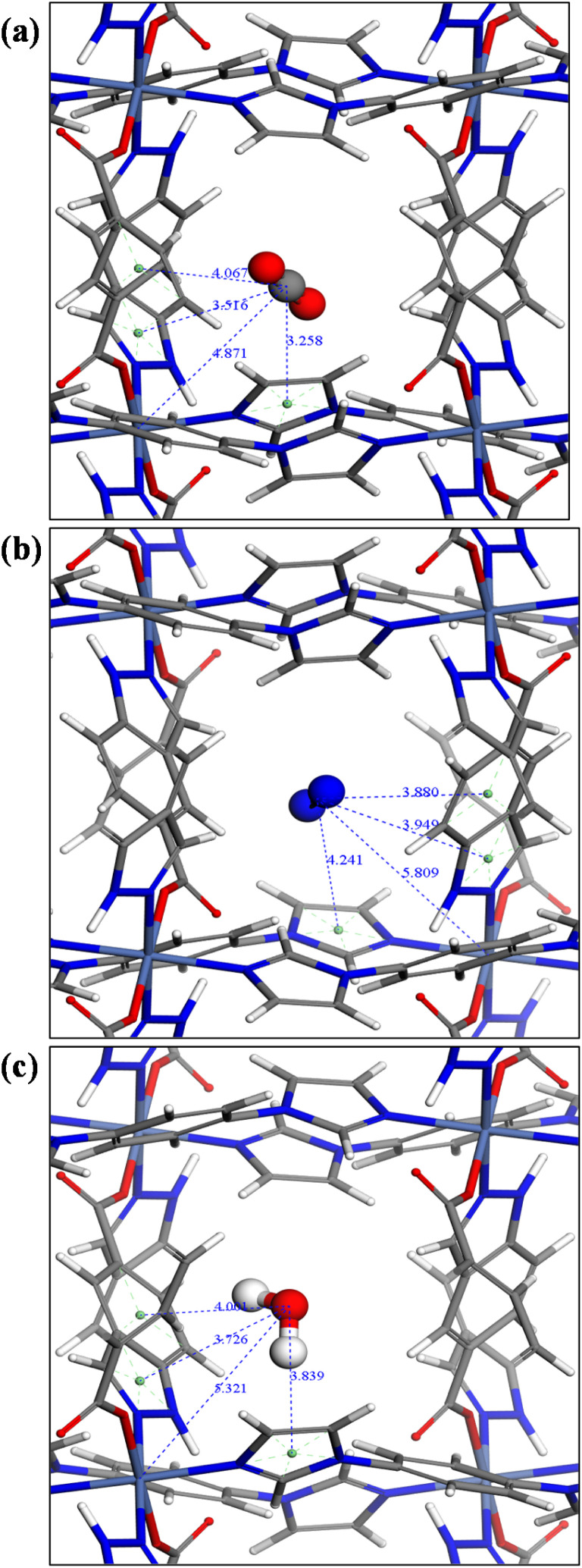
(a) CO_2_, (b) N_2_ and (c) H_2_O binding interactions observed by CMC simulations in pcu-1-Ni.

Analysis of the strongest adsorbate binding sites by CMC, in particular binding interactions and distances, further provides insight into preferential adsorption behaviour (Fig. 5). CO_2_ has a smaller kinetic diameter of 3.30 Å, compared to 3.64 Å for N_2_.^60^ The smaller kinetic diameter allows CO_2_ to bridge multiple binding sites simultaneously, enhancing its overall interaction strength. In Fig. 5a, the Ni–C(CO_2_) distance of 4.87 Å indicates the possibility of electrostatic interactions between the nickel centre and CO_2_. The distance between the centroid of the imidazole ring in the linker and C(CO_2_) is 3.26 Å, suggesting electrostatic forces or hydrogen bonding. Similarly, the distance between the centroid of the imidazole ring in purine and C(CO_2_) is 3.52 Å, indicative of π–π interactions. Additionally, the distance between the centroid of the pyrimidine ring of purine and C(CO_2_) is 4.07 Å, reflecting weaker van der Waals forces. In contrast, N_2_ has a slightly larger kinetic diameter which limits its ability to access these sites effectively, resulting in weaker adsorption. N_2_ indeed exhibits weaker binding interactions (Fig. 5b), the distance from Ni to the N_2_-centroid is 5.81 Å, signifying a reduction in electrostatic interaction strength compared to CO_2_. The distance between the centroid of the imidazole ring and the N_2_-centroid is 4.24 Å, while the distance between the centroid of the imidazole ring in purine and the centroid of N_2_ is 3.949 Å, both suggesting weaker π–π or van der Waals interactions. These longer distances also reflect the lower polarity of N_2_ compared to CO_2_, resulting in less favourable binding. In summary, CO_2_ demonstrates stronger binding interactions, driven by electrostatic and π–π interactions, while N_2_ binding is predominantly governed by weaker van der Waals interactions. For H_2_O (Fig. 5c), the Ni–H_2_O distance of 5.32 Å indicates weak electrostatic interactions between the nickel centre and the H_2_O molecule. The distance between the centroid of the imidazole ring in the linker and H_2_O is 3.84 Å, suggesting electrostatic or hydrogen bonding. The distance between the centroid of the imidazole ring in purine and H_2_O is 3.73 Å, indicative of π–π interactions. Additionally, the distance between the centroid of the pyrimidine ring of purine and H_2_O is 4.00 Å, reflecting weaker van der Waals forces. The water vapour sorption in pcu-1-Ni occurs through a pore filling mechanism that is influenced by the material's moderate hydrophobicity.

## Conclusions

In summary, this study introduces pcu-1-Ni, a new MOF derived by pillaring of sql nets through N-donor linkers. pcu-1-Ni exhibited strong CO_2_ adsorption and weak N_2_ adsorption, resulting in good CO_2_/N_2_ selectivity comparable to CO_2_/N_2_ selective sorbents other than HUMs. Computational studies provide insight into why pcu-1-Ni preferentially adsorbs CO_2_ over N_2_, validating its potential for CO_2_ capture. Additionally, pcu-1-Ni shows an S-shaped water vapour isotherm in the 45–65% RH range, making it suitable for indoor humidity control (IHC) applications. In addition, the low water uptake in the 0–50% RH range enhances its potential for flue gas separation, where moisture management is essential. This distinctive balance of strong CO_2_ selectivity, minimal water adsorption at low RH coupled with water uptake at optimal RH range for IHC is unusual. Whereas hydrolytic stability was found to be unsuitable for practical utility, we anticipate that pcu-1-Ni can serve as a prototype for the generation of a broader family of related sorbents through metal and/or pillar substitution to enable improvements to stability and separation performance.

## Author contributions

The manuscript was written through contributions of all authors. All authors have given approval to the final version of the manuscript. CRediT: S. M. S, A. A. B, A. C. E: conceptualization, investigation, methodology, writing-original draft, review and editing; L. C, T. H.: investigation, writing-review and editing; S. J. N, M. V: formal analysis, writing-review and editing; M. Z.: funding acquisition, formal analysis, writing-review and editing.

## Conflicts of interest

There are no conflicts to declare.

## Supplementary Material

TA-013-D5TA01995H-s001

TA-013-D5TA01995H-s002

TA-013-D5TA01995H-s003

TA-013-D5TA01995H-s004

TA-013-D5TA01995H-s005

## Data Availability

The data supporting the findings of this study are available in the ESI† or from the authors upon request.
