# Supplementary material for: A pcu topology metal–organic framework, Ni(1,4-bib)(inca)2, that exhibits high CO2/N2 selectivity and low water vapour affinity
Source: J Mater Chem A Mater. 2025 Apr 29;13(23):17562–8. doi: 10.1039/d5ta01995h (PMC12068445; doi:10.1039/d5ta01995h)
Supplement: TA-013-D5TA01995H-s001 [file TA-013-D5TA01995H-s001.pdf]

Supplementary Information (SI) for Journal of Materials Chemistry A.
This journal is © The Royal Society of Chemistry 2025

Electronic Supplementary Information (ESI)

A pcu Topology Metal-Organic Framework, Ni(1,4-bib)(inca)$_2$, that exhibits high CO_2/N_2 selectivity and low water vapour affinity.

Samuel M. Shabangu,[a] Alan C. Eaby,[a] Sousa Javan Nikkhah,[a] Lilia Croitor,[a] Tao He,[a] Andrey A. Bezrukov,[a] Matthias Vandichel[a] and Michael J. Zaworotko [a*]

[a] Department of Chemical Sciences, Bernal Institute, University of Limerick, Limerick V94 T9PX, Republic of Ireland

Table of Contents

Methods 3

S1. CSD survey 3

S2. Materials and synthesis 3

S3. Single crystal X-ray Diffraction Measurements 3

S4. Powder X-ray Diffraction Measurements 3

S5. Thermogravimetric Analysis 3

S6. Variable temperature Powder X-ray Diffraction Measurements 4

S7. Gas sorption Measurements 4

S8. IAST calculations 4

S9. Dynamic vapour sorption 4

S10. Computational Methods 5

References 16

Fig.S1: 3D **pcu** topology **pcu-1-Ni** with modelled DMF and H_2O solvents molecules........ 8

Fig.S2: PXRD diffractograms of calc. and experimental **pcu-1-Ni** 8

Fig.S3: TGA curves of **pcu-1-Ni** with loss of channel........ 9

Fig.S4: VT-PXRD diffractograms of **pcu-1-Ni** under N_2........ 9

Fig.S5: Atomic structures and point charges of H_2O, CO_2, and N_2........ 11

Fig.S6: Supercell of 2x2x2 of **pcu-1-Ni** with two pore types indicated: Type A and B........ 11

Fig.S7: Adsorption isotherms obtained for GCMC calculations for of CO_2 and N_2........ .. 14

Methods

S1. CSD survey

A survey using CSD (v5.44)[1] search was conducted on coordination networks sustained by inca and metal ion. The list of refcodes obtained was analysed using mercury[2] to assess guest molecules and assign dimensionality. Sorption results were also surveyed through published articles based on the refcode deposited (**Table S1**).

S2. Materials and synthesis

The reagents and solvents wee commercially available and were used without further purification.

Synthesis of **pcu-1-Ni**: A mixture of $Ni(NO_3)_2{\cdot}6H_2O$ (29 mg, 0.1 mmol), 1,4-bib (11 mg, 0.1mmol), inca (16 mg, 0.1 mmol) and DMF/H_2O (6/2 mL) was added to a 28 mL glass vial. The vial was capped tightly, ultrasonicated for 5 minutes and then placed in an oven at 120 °C. After 12 hours, the vial was removed from the oven and allowed to cool to room temperature. Block shaped crystals were harvested by filtration and washed with DMF. Yield: 96%

S3. Single crystal X-ray Diffraction Measurements

High quality single crystals of **pcu-1-Ni** were chosen for single crystal X-ray diffraction measurements. Diffraction data for **pcu-1-Ni** (170 K) was collected on a Bruker Quest diffractometer equipped with a IμS microfocus X-ray source (Cu Kα, λ = 1.54178 Å; Mo Kα, (λ = 0.71073 Å) and CMOS detector. In all cases, data was indexed, u8)=p['4ntegrated and scaled using Bruker SAINT software.[3] Space group determination was performed simultaneously with structure solution using SHELXT[3] intrinsic phasing methods through the X-Seed[4,5] graphical user interface. Zinc, carbon, nitrogen, oxygen and hydroxo hydrogen atoms of the host were refined anisotropically using SHELXL, using full-matrix least squares minimization.[4] Host hydrogen atomic positions were calculated using riding models. Selected crystallographic parameters are reported in Table S2 and S3. Pore volumes and geometries (pore limiting diameters and maximum pore diameters) were calculated using the Pore Analyzer (default settings) feature in Mercury.[2] Crystal structures were visualized using Mercury.[2] Difference electron density maps were calculated using OLEX2.[5]

S4. Powder X-ray Diffraction Measurements

Diffractograms were recorded using a PANalytical Empyrean™ diffractometer equipped with a PIXcel3D detector operating in scanning line detector mode with an active length of 4 utilizing 255 channels. The diffractometer is outfitted with an Empyrean Cu LFF (long fine-focus) HR (9430 033 7310x) tube operated at 40 kV and 40 mA and CuKα radiation (λα = 1.540598 Å) was used for diffraction experiments. Continuous scanning mode with the goniometer in the theta-theta orientation was used to collect the data. Incident beam optics included the Fixed Divergences slit with anti-scatter slit PreFIX module, with a 1/8° divergence slit and a 1/4° anti-scatter slit, as well as a 10 mm fixed incident beam mask and a Soller slit (0.04 rad). Divergent beam optics included a P7.5 anti-scatter slit, a Soller slit (0.04 rad) and a Ni-β filter. In a typical experiment, 25 mg of sample was dried, ground into a fine powder and was loaded on a zero background silicon disks. The data was collected from 5°–40° (2θ) with a step-size of 0.0131303° and a scan time of 30 seconds per step. Crude data were analyzed using the X'Pert HighScore Plus™ software V 4.1 (PANalytical, The Netherlands).

S5. Thermogravimetric Analysis

Thermogravimetric analyses (TGA) were performed under N_2 using a TA Instruments Q50 system. Samples were loaded into aluminium sample pans and heated at 283 K min^{-1} from room temperature to 773K.

S6. Variable temperature Powder X-ray Diffraction Measurements

Diffractograms at different temperatures were recorded using a PANalytical X'Pert Pro-MPD diffractometer equipped with a PIXcel3D detector operating in scanning line detector mode with an active length of 4 utilizing 255 channels. Anton Paar TTK 450 stage coupled with the Anton Paar TCU 110 Temperature Control Unit was used to record the variable temperature diffractograms. The diffractometer is outfitted with an Empyrean Cu LFF (long fine-focus) HR (9430 033 7300x) tube operated at 40 kV and 40 mA and CuKα radiation (λα = 1.54056 Å) was used for diffraction experiments. Continuous scanning mode with the goniometer in the theta-theta orientation was used to collect the data. Incident beam optics included the Fixed Divergences slit, with a 1/4° divergence slit and a Soller slit (0.04 rad). Divergent beam optics included a P7.5.

S7. Gas sorption Measurements

Before performing the gas sorption experiments, a freshly prepared sample of **pcu-1-Ni** were placed in a quartz tube and degassed under high vacuum using a Smart VacPrep instrument at 373 K for 10 h to remove any remaining solvent molecules. Isotherms were measured using a Micromeritics 3Flex sorption analyser. Gases were used as obtained from BOC Gases (Ireland), with the following certified purities: research-grade CO_2 (99.995%) N_2 (99.9995%%). Bath temperature of 195 K were maintained using dry ice–acetone slurry. The temperature at 77 K was maintained using a 4 L Dewar filled with liquid nitrogen. Bath temperatures of 273 and 298 K were precisely controlled with a Julabo ME (v.2) recirculating control system containing a mixture of ethylene glycol and water.

S8. IAST calculations

The selectivities for the adsorbate mixture composition of interest were calculated from the single component adsorption isotherms using Ideal Adsorbed Solution Theory (IAST).[6, 7] Single component adsorption isotherms for CO_2 and N_2 at 298 K were fitted to the Langmuir equation:

(P) = $q * kP$ 1 + kP 11

where: $n(P)$ is the uptake (mmol/g) as a function of pressure

P is the total pressure (bar) of the bulk gas at equilibrium with the adsorbed phase

q is the saturation uptake (mmol/g)

k is the affinity coefficient (bar^{-1})

Then, mixed-gas fractional uptakes were calculated and the selectivity was obtained as follows:

Si/j = (xi/xj)/ (yi/yj)

xi and xj are the mole fractions of components i and j in the adsorbed phase yi and yj are the mole fractions of components i and j in the gas phase.

Fitting parameters for the CO_2, N_2 (collected at 298 K) are listed in **Table S4**.

S9. Dynamic vapour sorption

Water vapor sorption isotherm measurements were performed using Adventure dynamic vapor sorption (DVS) instrument manufactured by Surface Measurement Systems. The instrument gravimetrically measures water vapor uptake using air as a carrier gas. Digital mass flow controllers regulate flows of dry and saturated gases. Relative humidity is generated by precisely mixing dry and saturated gas flows in desired flow ratios which produce expected relative humidity. Pure water was used to generate water vapor for these measurements and temperature was maintained at 300 K by enclosing the

system in a temperature-controlled incubator. The mass of the sample was determined by a high-resolution microbalance with a precision of 0.01 μg. Microbalance has symmetric configuration with two branches of the balance being exposed to the same gas and being kept at the same temperature, which allows negation of buoyancy and drag effects. Instrument allows measurement of 2 samples in parallel. Prior to the measurement, sample was in-situ activated in dry air at 373 K using built-in preheater and consequently cooled to sorption temperature. Isotherm measurements were performed on approximately 9 mg of sample powder. 400 sccm min^{-1} total flow was used for the measurements at 300 K. The flow is split between two samples. For each isotherm point, dm/dt < 0.01 %/min was used as criteria of reaching equilibrium.

Water vapor sorption cycling was performed at 300 K on a Surface Measurement Systems DVS adventure instrument using air as a carrier gas to gravimetrically measure the uptake and loss of vapor. The mass of the sample was determined by comparison to an empty reference pan and recorded by a high-resolution microbalance with a precision of 0.1 μg. Prior to the measurement, the sample (9 mg) was activated in dry air at 373 K for 120 minutes. Humidity swing was measured between two points 0 and 85 % RH. 50 cycles were subsequently performed.

Experimental sorption kinetics was measured on DVS adventure instrument using humidity swing experiment performed between two points for 0-85 %RH on a 9 mg sample.

S10. Computational Methods

Periodic Density Functional Theory calculations were performed using BEEF-vdW[8] exchange-correlation functional to optimize the atomic positions of the framework before the Monte Carlo simulations (see further). In particular, the projected augmented wave (PAW) formalism[9] was employed as implemented in the Vienna Ab Initio Simulation Package (VASP 6.4.2)[10] and was used with standard PAW potentials. The atomic positions were optimized within their experimental unit cells (for cell parameters of **pcu-1-Ni** see **Table S5**) using the conjugate gradient algorithm with force and electronic convergence criteria of 0.01 eV/Å and 10^{-6} eV, Gaussian smearing of 0.05 eV, a plane wave energy cut-off of 600 eV, 2x2x2 Monkhorst-Pack[11] k-point grids, and with the assumption of two unpaired electrons per Ni atom.

Canonical Monte Carlo (CMC) simulations[12] were performed to confirm the main binding site locations for H_2O, CO_2, and N_2 in **pcu-1-Ni** framework. CMC simulations were performed in Materials Studio at 300 K for water and at 298 K for CO_2, and N_2 on a 2x2x2 supercell of **pcu-1-Ni**. To describe the electrostatics upon adsorption, the atomic point charges of the frameworks and the adsorbate were determined via the charge equilibration (Qeq) method.[13] The point charges used for H_2O, CO_2, and N_2 are given in **Figure S5.** Furthermore, the Lennard-Jones parameters were obtained from the Universal Force Field (UFF)[14] as implemented in the Material Studio forcefield library, employing the Lorentz-Berthelot mixing rule for interactions between unlike atoms. A cut-off distance of 12 Å was applied for non-bonded interactions, with the potentials smoothly truncated using a cubic spline function over a spline width of 1 Å. Electrostatic interactions were calculated using the Ewald[15] summation method for enhanced accuracy.

For the CMC simulations, the framework was considered rigid with atoms fixed at their DFT-optimized positions. The CMC simulations were performed at a fixed loading of one sorbate molecule (per supercell). In the canonical ensemble, the Metropolis sampling method considered different moves, such as translation (corresponds with a translation of the selected adsorbate molecule), rotation around the center of mass of the selected adsorbate molecule, regrowth (removes a selected adsorbate molecule from the system and reintroduces it at a random position with random orientation), and conformer (collects multiple adsorbate conformations), with relative probabilities of 1, 1, 0.1 and 1, respectively. CMC simulations included 1×10^6 loading steps, followed by 1×10^6 equilibration steps, and finally, 1×10^6 production steps to ensure reasonable ensemble averages. The output of the CMC simulations was visualized as adsorbate density fields, encompassing the mass-middle points of all successful adsorbate MC moves (see **Tables S6**). The framework contains two types of pores, labelled as

Type A and Type B (see **Figure S6**). The density fields (**Table S6**) show pore type B contains more favourable adsorption sites than pore type A. We also created an isosurface of constant density and coloured it by the potential energy (see **Tables S7**).

Grand Canonical Monte Carlo (GCMC) simulations were performed to provide more insight into the adsorption isotherms of CO_2 and N_2. We refer to the above CMC section for details on the force field parameters and Metropolis sampling technique. The adsorption isotherms were calculated for the pressure range of 0 to 1 bar. Each GCMC simulation included 1 $\times 10^6$ equilibration steps, followed by 1 $\times 10^6$ production steps to ensure reasonable ensemble averages.

Furthermore, we conducted hybrid grand canonical Monte Carlo/Molecular Dynamics (GCMC/MD) simulations. This approach allowed us to investigate H_2O, CO_2 and N_2 adsorption within **pcu-1-Ni**, taking dynamic aspects into consideration. Hybrid GCMC/MD simulations were implemented for various pressures between 0 and 1 bar for CO_2 and N_2 at 298K (0.001, 0.03, 0.05, 0.10, 0.15, 0.20, 0.25, 0.30, 0.35, 0.40, 0.45, 0.50, 0.55, 0.60, 0.65, 0.70, 0.75, 0.80, 0.85, 0.90, 0.95, 1 bar) on a 2x2x2 supercell. The hybrid simulations for water were conducted across a range of relative humidities from 0% to 100% at a temperature of 300 K. At each pressure, the simulation starts with a GCMC run, including 1×10^6 MC equilibration and 1×10^6 MC production steps. Five low-energy configurations were randomly collected from the GCMC simulation at each pressure. Among these, the configuration with the lowest energy was selected as the input for the subsequent MD simulation. Following the GCMC runs, the selected configuration was equilibrated via MD for 500 ps (5×10^5 number of steps) in the NVT ensemble (constant number of particles, volume, and temperature). The Ni atoms in **pcu-1-Ni** were constrained to stabilize the framework structure and prevent it from disintegrating during the MD simulations. During MD, the temperature was maintained (at 298 for CO_2 and N_2 and 300 K for H_2O) by a Nosé-Hoover thermostat, while the adsorbates' atomic positions were updated according to the velocity Verlet scheme with a time step of 1 fs. Data was sampled at 20 frames during the MD simulations, each separated by 500 ps (5×10^5 number of steps). From these frames, the one with the lowest energy was selected and used as the input structure for the subsequent GCMC calculations at the next pressure level. A key distinction between our hybrid GCMC/MD simulation approach and the traditional GCMC method is that our approach accounts for atom dynamics and diffusion in addition to the adsorption phenomenon. Supplementary files in the form of **movie S1** (**pcu-1-Ni CO2**.mp4), **movie S2** (**pcu-1-Ni_N2**.mp4), and **movie S3** (**pcu-1-Ni _H2O**.mp4) showcasing the GCMC/MD simulations are provided for a more detailed visual understanding of the process. Consistent with the density maps from CMC simulations presented in **Tables S6** and **S7**, our GCMC/MD simulations indicate that pore type B is more favourable for all three adsorbates.

Table S1. Reported examples based on inca in the CSD

REFCODE	Linker type	Dimension	Sorption	Reference
RUHKEN	single	2D	n/a	[16]
RUHKEN01	single	2D	N_2 (77 K)	[17]
ZIJHUZ	single	2D	N_2 (77 K)	[17]
ZIJJEL	single	2D	n/a	[17]
BUTZUO	mixed	3D	CO_2 273 K	[18]
BUVBAY	mixed	3D	CO_2 273 K	[18]
ELENOB	single	3D	N_2 (77 K), CO_2, CH_4, C_2H_2, C_2H_4, C_2H_6 (273, 298 K)	[19]
SEGBOX	Single	3D	CO_2 (258, 273, 298 K)	[20]
NUYNED	single	2D	n/a	[16]
SEGBIR	single	2D	N_2 (77 K), CO_2 (258, 273 ,298 K), CH_4 (298 K)	[20]

Table S2: Selected crystallographic details of **Pcu-1-Ni**

Unit Cell Parameters	**Pcu-1-Ni**
Formula	$C_{28}H_{20}N_8Ni O_4,H_2O,C_3H_7$ NO
Formula weight (g/mol)	417.23
Temperature (K)	170 K
Crystal system	Monoclinic
Space group	*P*2$_1$/*c*
a (Å)	13.6323(7)
b (Å)	16.4865(8)
c (Å)	14.5470(7)
β (°)	101.815(2)
V (Å^3)	3200.16
Z	4
GooF	1.094
R_1 *[I > 2σ(I)]*	0.0355
WR_2 *[all data]*	0.1383

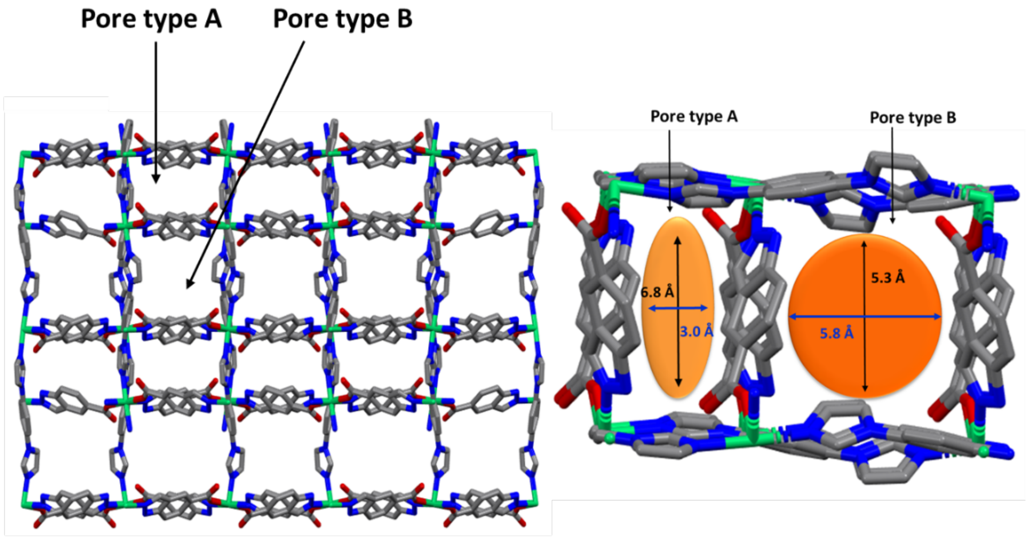

Figure S1: 3D **pcu** topology MOF structure of **pcu-1-Ni** with modelled DMF and H_2O solvent molecules and pore dimensions.

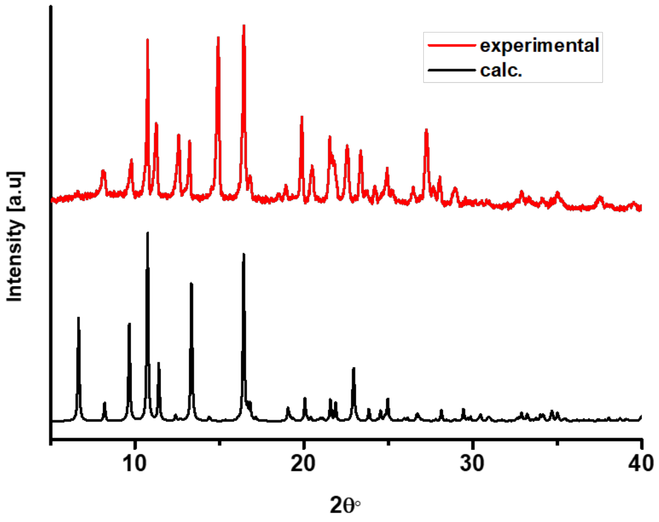

Figure S2: PXRD diffractograms of calc. and experimental **pcu-1-Ni**.

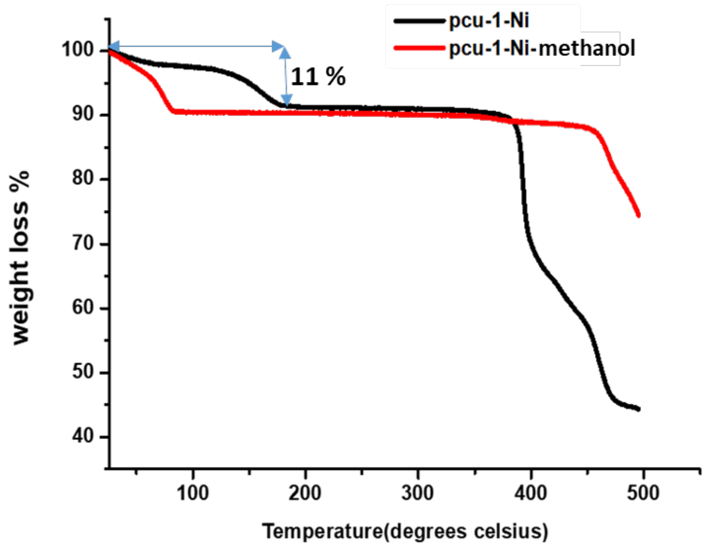

Figure S3: TGA curves of **pcu-1-Ni** and **pcu-1-Ni-methanol.**

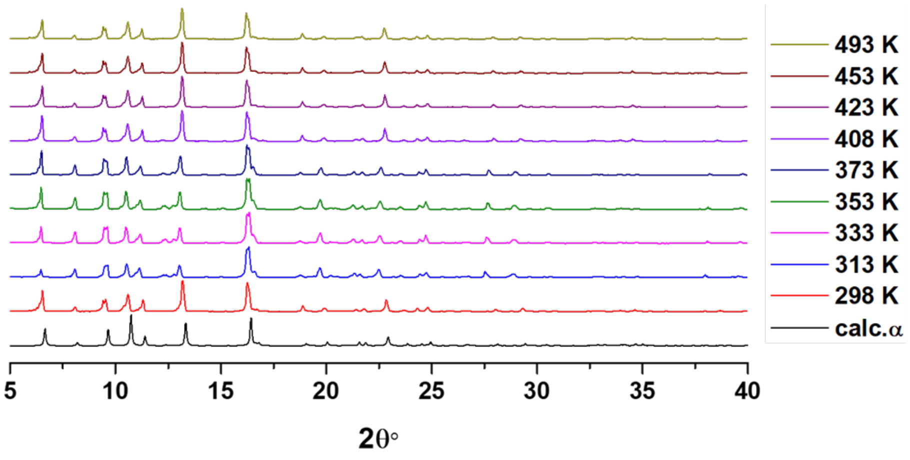

Figure S4: VT-PXRD diffractograms of **pcu-1-Ni** under N_2.

Table S3: Comparison of the adsorption characteristics of **pcu-1-Ni** with some of the leading CO_2 capturing MOMs.

MOF	CO_2 capacity at 298 K (mmol/g)		References
	0.15 bar	1 bar	
IISERP-MOF28	2.2	3.1	21
Calf-20 (303 K)	2.5	3.54	22
ALF	2.7	3.98	23
Zn-Atz_OX (293 K)	2.1	3.65	24
IISERP-MOF2 (303 K)	1.6	4.1	25
ZnF(daTZ)	0.96	3.3	26
mmen-Mg2 (dobpdc)	3.2	3.9	27
Zn3 (Atz)3 (PO4) (273K)	1.76	3.16	28
UTSA-16	2.54	4.4	29
MAF66	1.29	4.41	30
JUC-132-Cd	0.65	1.71	31
bio-MOF-12	1.34	3.15	32
bio-MOF-13	1.0	2.0	32
CAU-1	1.1	4.0	33
MIL-91(Ti)	1.5	3.5	34
NH2 -MIL-53(Al)	0.9	1.5	35
MIL-101(Cr)	0.6	2.25	36
SIFSIX-3-Cu	2.45	2.6	37
SIFSIX-3-Zn	2.38	2.55	37
Pcu-1-Ni	0.92	4.5	This work

Table S4: Isotherm fitting parameters and fit values for IAST calculations.

Adsorbent	Adsorbate	Model	R^2	q (mmol/g)	K (bar^{-1})
pcu-1-Ni	CO_2	Langmuir	0.999996	4.66162	2.09997
	N_2	langmuir	0.999228	0.729511	0.128511

Table S5: Cell parameters of **pcu-1-Ni**, total volume and applied k-point mesh during geometry optimization.

Structures	a (Å)	b (Å)	c (Å)	α (°)	β (°)	γ (°)	Volume (Å³)	k-point mesh
**pcu-1-Ni**	13.6143	16.4688	14.6303	90.00	102.842	90.00	11641.65	2x2x2

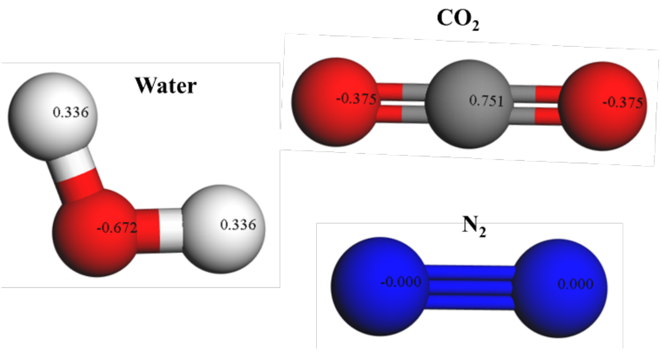

Figure S5: Atomic structures and point charges of H_2O, CO_2, and N_2.

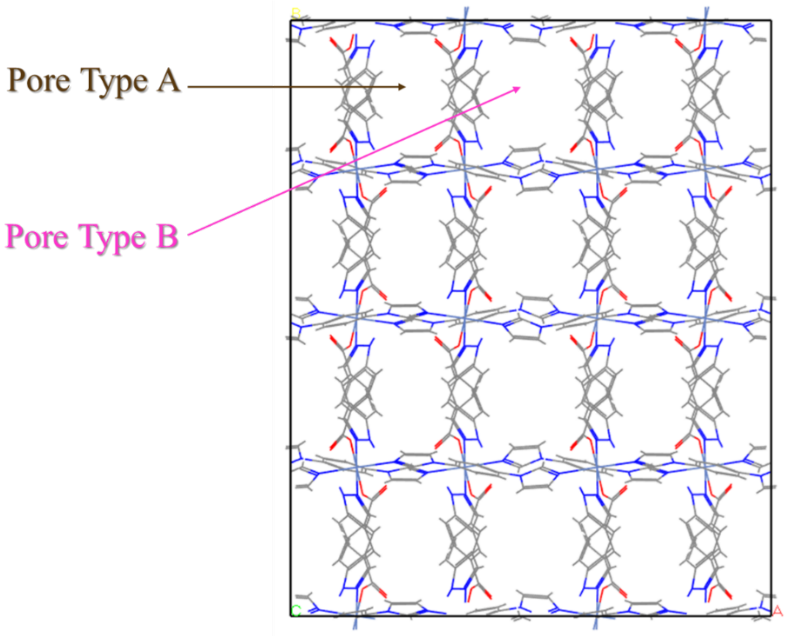

Figure S6: Supercell of 2x2x2 of **pcu-1-Ni** with two pore types indicated: Type A and B.

Table S6: The adsorbate density fields obtained from CMC simulations for water, CO_2, and N_2 at 298 K. They allow the identification of optimal binding sites. The colour map values indicate the density of the adsorbate in molecules/Å^3.

**Adsorbate**	Adsorbate density field in Ni(1,4-bib)(Inca)$_2$
**H_2O**	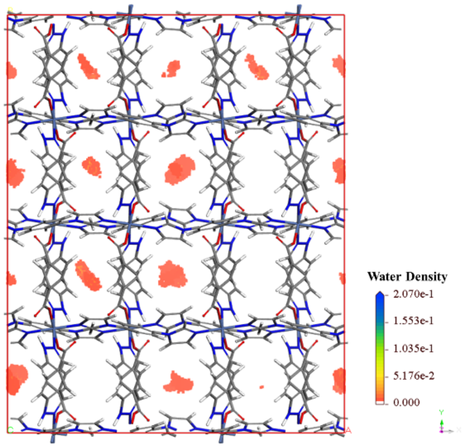
**CO_2**	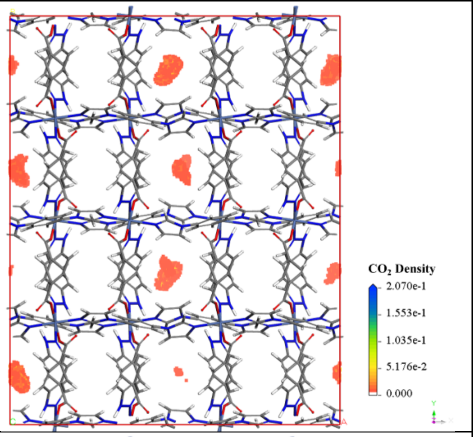
**N_2**	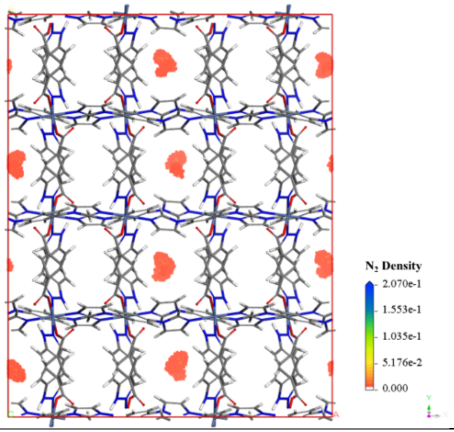

Table S7: The columns represent a display that combines the energy and density distribution information at 298 K. It has created an isosurface of constant density (isovalue= 0.00004) and coloured it by the potential energy. In this representation, regions shaded in dark blue signify the lowest energy, while those shaded in dark red denote the highest energy. This analysis can be used to identify favourable binding sites within the framework. The colour map values indicate the potential energy of the adsorbate in kcal/mol.

Adsorbate	
H_2O	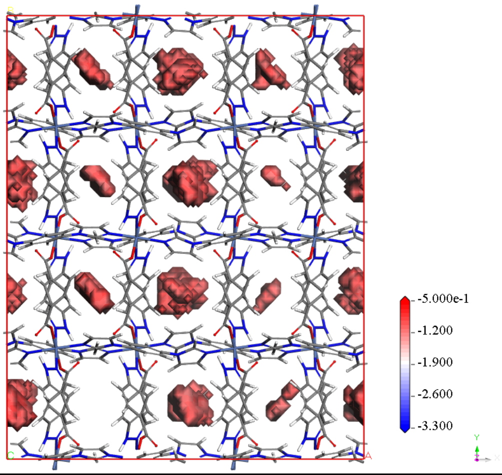
CO_2	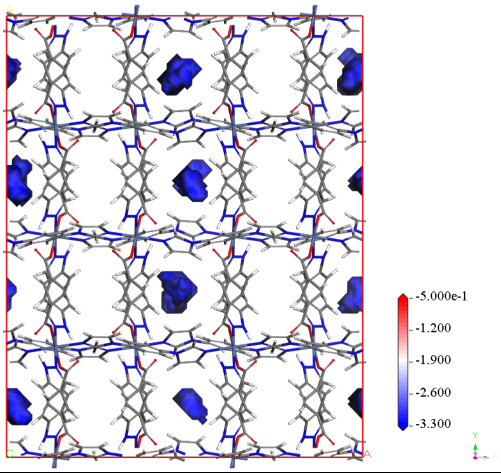
N_2	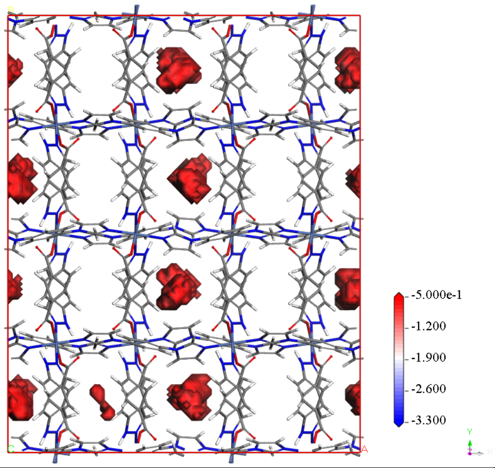

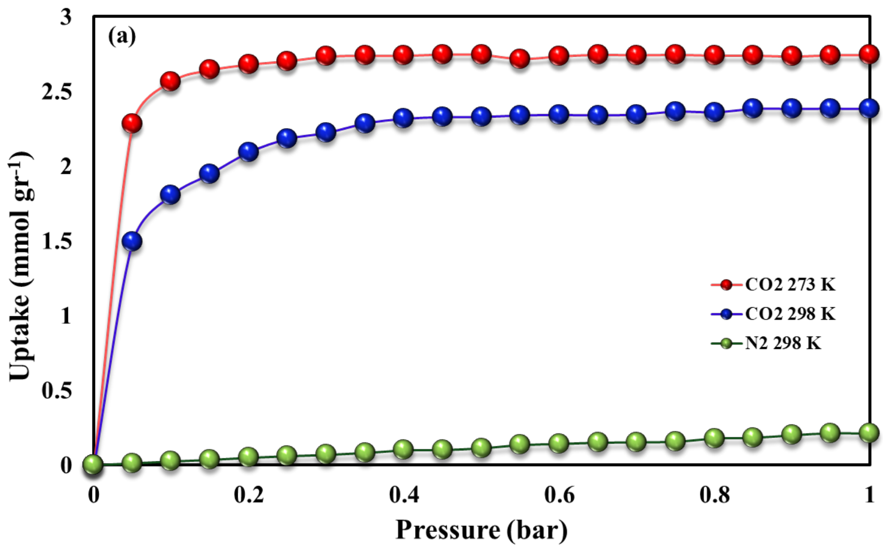

Figure S7: Adsorption isotherms obtained for GCMC calculations for of CO_2 and N_2

References

1. I. J. Bruno, J. C. Cole, P. R. Edgington, M. Kessler, C. F. Macrae, P. McCabe, J. Pearson and R. Taylor, *Acta Crystallographica Section B*, 2002, **58**, 389-397.
2. C. F. Macrae, I. Sovago, S. J. Cottrell, P. T. A. Galek, P. McCabe, E. Pidcock, M. Platings, G. P. Shields, J. S. Stevens, M. Towler and P. A. Wood, *Journal of Applied Crystallography*, 2020, **53**, 226-235.
3. T. Francart, A. van Wieringen and J. Wouters, *J.Neurosci.Meth.*, 2008, **172**, 283-293.
4. G. M. Sheldrick, *Acta Crystallographica Section C Structural Chemistry*, 2015, **71**, 3-8.
5. O. V. Dolomanov, L. J. Bourhis, R. J. Gildea, J. A. K. Howard and H. Puschmann, *Journal of Applied Crystallography*, 2009, **42**, 339-341.
6. A. L. Myers and J. M. Prausnitz, *AIChE Journal*, 1965, **11**, 121-127.
7. K. S. Walton and D. S. Sholl, *AIChE Journal*, 2015, **61**, 2757-2762.
8. J. Wellendorff, K. T. Lundgaard, A. Møgelhøj, V. Petzold, D. D. Landis, J. K. Nørskov, T. Bligaard and K. W. Jacobsen, *Physical Review B*, 2012, **85**.
9. P. E. Blöchl, *Physical Review B*, 1994, **50**, 17953-17979.
10. G. Kresse and J. Furthmüller, *Computational Materials Science*, 1996, **6**, 15-50.
11. H. J. Monkhorst and J. D. Pack, *Physical Review B*, 1976, **13**, 5188-5192.
12. D. Frenkel and B. Smit, *Understanding molecular simulation: from algorithms to applications*, Elsevier, 2023.
13. T. R. Lucas, B. A. Bauer and S. Patel, *Bba-Biomembranes*, 2012, **1818**, 318-329.
14. *Journal*.
15. A. Y. Toukmaji and J. A. Board, *Computer Physics Communications*, 1996, **95**, 73-92.
16. A. A. García-Valdivia, A. García-García, F. Jannus, A. Zabala-Lekuona, J. M. Méndez-Arriaga, B. Fernández, M. Medina-O'donnell, G. B. Ramírez-Rodríguez, J. M. Delgado-López, L. M. Pastrana-Martínez, J. Cepeda, J. A. Lupiáñez, F. J. Reyes-Zurita and A. Rodríguez-Diéguez, *Journal of Inorganic Biochemistry*, 2020, **208**, 111098.
17. X. Liang, S. Wang, J. Feng, Z. Xu, Z. Guo, H. Luo, F. Zhang, C. Wen, L. Feng, C. Wan and M.-M. Titirici, *Inorganic Chemistry Frontiers*, 2023, **10**, 2961-2977.
18. A. A. García-Valdivia, M. Pérez-Mendoza, D. Choquesillo-Lazarte, J. Cepeda, B. Fernández, M. Souto, M. González-Tejero, J. A. García, G. M. n. Espallargas and A. Rodríguez-Diéguez, *Cryst. Growth Des.*, 2020, **20**, 4550-4560.

19. W.-F. Zhang, Y. Du, X.-Y. Sun, H.-M. Pan, Y.-Y. Ma, D.-Y. Li, S. Wu, T. Yan and Z.-H. Jing, *Inorganic Chemistry Communications*, 2021, **126**, 108469.
20. C. S. Hawes, R. Babarao, M. R. Hill, K. F. White, B. F. Abrahams and P. E. Kruger, *Chem. Commun.*, 2012, **48**, 11558-11560.
21. P. Singh, H. D. Singh, A. H. Menon and R. Vaidhyanathan, *Chem. Commun.*, 2023, **59**, 5559-5562.
22. J. B. Lin, T. T. T. Nguyen, R. Vaidhyanathan, J. Burner, J. M. Taylor, H. Durekova, F. Akhtar, R. K. Mah, O. Ghaffari-Nik, S. Marx, N. Fylstra, S. S. Iremonger, K. W. Dawson, P. Sarkar, P. Hovington, A. Rajendran, T. K. Woo and G. K. H. Shimizu, *Science*, 2021, **374**, 1464-+.
23. H. A. Evans, D. Mullangi, Z. Deng, Y. Wang, S. B. Peh, F. Wei, J. Wang, C. M. Brown, D. Zhao, P. Canepa and A. K. Cheetham, *Sci.Adv.*, 2022, **8**, eade1473.
24. R. Vaidhyanathan, S. S. Iremonger, G. K. H. Shimizu, P. G. Boyd, S. Alavi and T. K. Woo, *Science*, 2010, **330**, 650-653.
25. S. Nandi, S. Collins, D. Chakraborty, D. Banerjee, P. K. Thallapally, T. K. Woo and R. Vaidhyanathan, *J. Am. Chem. Soc*, 2017, **139**, 1734-1737.
26. Z. Shi, Y. Tao, J. Wu, C. Zhang, H. He, L. Long, Y. Lee, T. Li and Y.-B. Zhang, *J. Am. Chem. Soc*, 2020, **142**, 2750-2754.
27. T. M. McDonald, W. R. Lee, J. A. Mason, B. M. Wiers, C. S. Hong and J. R. Long, *J. Am. Chem. Soc*, 2012, **134**, 7056-7065.
28. R. Vaidhyanathan, S. S. Iremonger, G. K. H. Shimizu, P. G. Boyd, S. Alavi and T. K. Woo, *Angewandte Chemie*, 2012, **124**, 1862-1865.
29. A. Masala, J. G. Vitillo, G. Mondino, C. A. Grande, R. Blom, M. Manzoli, M. Marshall and S. Bordiga, *ACS Applied Materials & Interfaces*, 2017, **9**, 455-463.
30. R.-B. Lin, D. Chen, Y.-Y. Lin, J.-P. Zhang and X.-M. Chen, *Inorganic Chemistry*, 2012, **51**, 9950-9955.
31. H. He, Y. Song, C. Zhang, F. Sun, R. Yuan, Z. Bian, L. Gao and G. Zhu, *Chem. Commun.*, 2015, **51**, 9463-9466.
32. T. Li, D.-L. Chen, J. E. Sullivan, M. T. Kozlowski, J. K. Johnson and N. L. Rosi, *Chemical Science*, 2013, **4**, 1746-1755.
33. X. Si, C. Jiao, F. Li, J. Zhang, S. Wang, S. Liu, Z. Li, L. Sun, F. Xu, Z. Gabelica and C. Schick, *Energy & Environmental Science*, 2011, **4**, 4522-4527.
34. V. Benoit, R. S. Pillai, A. Orsi, P. Normand, H. Jobic, F. Nouar, P. Billemont, E. Bloch, S. Bourrelly, T. Devic, P. A. Wright, G. de Weireld, C. Serre, G. Maurin and P. L. Llewellyn, *J.Mater.Chem.A*, 2016, **4**, 1383-1389.
35. S. Couck, J. F. M. Denayer, G. V. Baron, T. Rémy, J. Gascon and F. Kapteijn, *J. Am. Chem. Soc*, 2009, **131**, 6326-6327.
36. Z. Zhou, L. Mei, C. Ma, F. Xu, J. Xiao, Q. Xia and Z. Li, *Chemical Engineering Science*, 2016, **147**, 109-117.
37. O. Shekhah, Y. Belmabkhout, Z. Chen, V. Guillerm, A. Cairns, K. Adil and M. Eddaoudi, *Nat.Commun.*, 2014, **5**, 4228.
